# Ultrasensitive Peptide-Based Electrochemical Biosensor for Universal Diagnostic of Dengue

**DOI:** 10.3390/bios15040236

**Published:** 2025-04-08

**Authors:** Isis Campos Prado, João Pedro Rangel da Silva Carvalho, André Souza Araujo, Paloma Napoleão-Pêgo, Salvatore Giovanni De-Simone

**Affiliations:** 1 Center for Technological Development in Health (CDTS)/National Institute of Science and Technology for Innovation in Neglected Population Diseases (INCT-IDPN), Oswaldo Cruz Foundation (FIOCRUZ), Rio de Janeiro 21040-900, RJ, Brazil; joaopedrorsc@gmail.com (J.P.R.d.S.C.); paloma.pego@fiocruz.br (P.N.-P.); 2 Program of Post-Graduation on Science and Biotechnology, Biology Institute, Federal Fluminense University, Niteroi 24020-141, RJ, Brazil; 3 Analytical Chemistry Department, Chemistry Institute, Federal Fluminense University, Niteroi 24020-141, RJ, Brazil; araujoas@outlook.com; 4 Epidemiology and Molecular Systematics Laboratory (LEMS), Oswaldo Cruz Institut, Oswaldo Cruz Foundation (FIOCRUZ), Rio de Janeiro 21040-900, RJ, Brazil; 5 Program of Post-Graduation on Parasitic Biology, Oswaldo Cruz Institute, Oswaldo Cruz Foundation, Rio de Janeiro 21040-900, RJ, Brazil

**Keywords:** dengue, IgG epitope, serologic diagnostic, electrochemistry, voltammetric biosensor

## Abstract

Dengue is a neglected disease mainly affecting tropical and subtropical countries. The diagnosis of dengue fever is still a problem since most of it is made from whole or recombinant DENV proteins, which present cross-reactions with other members of the Flavivirus family. Therefore, there is still a huge demand for new diagnostic methods that provide rapid, low-cost, easy-to-use confirmation. Thus, in this study, we developed an affordable electrochemical biosensor for rapidly detecting immunoglobulin G (IgG) serological antibodies in the sera of DENV-infected patients. An identified linear B-cell epitope (DENV/18) specific for DENV 1–4 serotypes recognized by IgG in patient sera was selected as a target molecule after a microarray of peptides using the SPOT-synthesis methodology. After chemical synthesis, the DENV/18-peptide was immobilized on the surface of the working electrode of a commercially available screen-printed gold electrode (SPGE). The capture of DENV-specific IgG allowed for the formation of an immunocomplex that was measured by cyclic voltammetry (CV) and differential pulse voltammetry (DPV) using a potassium ferrocyanide/ferricyanide ([Fe(CN)_6_]^3−/4−^) electrochemical probe. An evaluation of the biosensor’s performance showed a detection limit of 100 µg mL^−1^ for the synthetic peptides (DENV/18) and 1.21 ng mL^−1^ in CV and 0.43 ng mL^−1^ in DPV for human serum, with a sensitivity of 7.21 µA in CV and 8.79 µA in DPV. The differentiation of infected and uninfected individuals was possible even at a high dilution factor that reduced the required sample volumes to a few microliters. The final device proved suitable for diagnosing DENV by analyzing real serum samples, and the results showed good agreement with molecular biology diagnostics. The flexibility to conjugate other antigenic peptides to SPEs suggests that this technology could be rapidly adapted to diagnose other pathogens.

## 1. Introduction

Dengue virus (DENV) infection is distributed worldwide, with an estimated 390 million new cases and up to 36,000 deaths per year worldwide [1,2]. Between 2.5 and 3 billion people live in risk conditions in countries where dengue is endemic [3]. The most affected regions are Africa, the Americas, Southeast Asia, the Eastern Mediterranean, and the Western Pacific [4]. This highlights the challenges of diagnosing viral infections such as dengue, particularly in areas far from clinical laboratories.

Dengue virus (DENV) is a single-stranded RNA virus of the genus flavivirus. It is transmitted primarily by the *Aedes aegypti* mosquito. The etiological agent of DENV is characterized by four antigenically and phylogenetically distinct serotypes (DENV-1, DENV-2, DENV-3, and DENV-4), within which there is considerable intra-serotype genetic variation. In recent years, dengue virus has been detected using antigen-based rapid diagnostic tests, virus isolation, and molecular tools [5,6,7]. However, antigen detection using naïve or recombinant large proteins has low sensitivity and specificity, principally due to the multiple cross-reactivity with other organisms [8,9,10,11,12,13]. Therefore, molecular diagnostic tools, such as reverse transcription polymerase chain reaction (RT-PCR), are increasingly applied in detecting DENV [14,15]. Unfortunately, such techniques are expensive and have high technical requirements, which limits their wide application [16]. An ideal diagnostic tool should have high sensitivity, specificity, flexibility, and simplification [17,18,19].

In recent years, synthetic peptides have become attractive elements for biorecognition in the construction of biosensors [20,21] due to their characteristics such as high stability against denaturation and ease of modification, simple acquisition, specificity, cost-effectiveness, standard synthetic protocol, chemical combination, and random library selection. For example, peptides with short amino acid chains generally have better chemical and conformational stability than proteins [22]. The appropriate use of peptides as biorecognition molecules in biosensors is intrinsically associated with their hydrophobic or hydrophilic nature, total charge obtained from the isoelectric point, storage conditions, and solubility in different buffers, which optimizes the conditions for the application of biosensors. Unfortunately, a measurable signal cannot be directly obtained from peptides in response to a binding. Therefore, conjugation with a signal marker is needed, which is an efficient strategy for converting the analyte information into a measurable signal [23]. Thus, the unique peptide properties are widely applied in developing new peptide-based electrochemical biosensors (PBEBs) for detecting a broad spectrum of proteins [24,25]. Peptide-based biosensors exhibit excellent properties, enabling quantitative preparation and long-term storage of biosensors. They play a crucial role in developing modern electrochemical biosensors for diverse applications in clinical diagnostics, biosafety, chemical and biological analysis, environmental monitoring, and healthcare [26,27].

Recently, our group has been working on developing new peptides for DENV. New serological marker assembly technology maps more antigenic epitopes selected from immunoreactivity tests for different serotypes. This study has enabled us to obtain more specific antigenic peptides than currently available, representing a major advance in diagnosing DENV in its early and late stages. Thus, using these peptides in new sensor platforms may be valuable in sera epidemiological studies to differentiate DENV infection or vaccination against related flaviviruses [28,29,30] and COVID [31,32]. This becomes even more important as this virus increasingly presents an epidemic-endemic profile in Brazil.

Rapid tests based on biosensors have been identified as one of the most attractive analytical possibilities as they are practical, fast, and versatile and were developed to simplify the testing process, reducing costs and shortening analysis time. One of the important steps in constructing a biosensor is immobilizing the biological material on the sensor surface [33]. L-cysteine is often used in gold electrodes due to its ability to form a self-donor film, improving the electroactivity and selectivity of the electrode. In addition, cysteine helps stabilize the electrode surface, facilitating the detection of analytes at low concentrations. Thus, the technique is especially useful in biosensors and analytical applications [34,35]. L-cysteine on gold electrodes is mainly used in biosensors to detect biomolecules such as amino acids, proteins, and heavy metals. Acting as a mediator, it improves electron transfer and increases detection sensitivity. In addition, cysteine can be used in studies of biomolecular interactions, allowing for the analysis of bonds between proteins and other molecules. This makes it valuable in medical diagnostics and environmental monitoring [36].

This study consists of the construction of an electrochemical biosensor using ultra-specific synthetic peptides (epitopes) for DENV 1–4 serotypes; a L-cysteine solution is used for the immobilization of the epitope on the surface of the printed gold electrode, and a glutaraldehyde solution is used to form a crosslink to fix the structures [37]. The current was generated by the reduction of electroactive species with a potassium ferrocyanide/ferricyanide solution applied to the electrode surface, monitored by the oxidation potential obtained for the ([Fe(CN)_6_]^3−/4−^) electrochemical probe. The magnitude of the current, used for analysis at the electrode/solution interface, such as the ion behavior or structure of the electrical double layer, was then related to the amount of analyte present. Since the peptide (epitope) and the circulating antibody in human serum are not intrinsically electroactive, a suitable label must be introduced to promote an electrochemical reaction in the biosensor [38]. In this study, the interaction was monitored using the redox reaction of the electrochemical probe.

In this context, the electrochemical biosensor was constructed from a competitive assay in which the specific antibody dispersed in the serum solution competes for a limited number of epitope binding sites. The electroanalytical response was obtained through the electrochemical probe. The electric current signal was measured when the potential was applied to the working electrode. From the decrease in the current signal, due to the physical blockage on the electrode surface, it was possible to observe and measure the level of the interaction between the DENV epitope and antibodies in human serum. The biosensor presented a detection limit of 1.21 ng mL^−1^ and 0.43 ng mL^−1^ and a coefficient of variation of 8.04% and 4.06% in CV and DPV, respectively.

## 2. Materials and Methods

### 2.1. Materials

Potassium ferrocyanide [K_4_Fe(CN)_6_] and potassium ferricyanide [K_3_Fe(CN)_6_] were obtained from (Sigma-Merck, St Luis, MO, USA). Phosphate-buffered saline solutions (PBS, pH 7.4) were obtained by mixing 0.1 mmol L^−1^ NaH_2_PO_4_, Na_2_HPO_4_, KCl, and 13 mmol L^−1^ NaCl. L-cysteine (L-Cys), glutaraldehyde (GA) solution (2.5% *w*/*v*), and other chemicals were obtained from (Sigma-Merck, St. Louis, MO, USA). All the solutions were prepared with deionized water (>18.1 MΩ cm) obtained from a Nanopure Diamond system (Barkstead, Dubuque, IA, USA).

### 2.2. Patient Samples and Project Approval

A total of 24 human sera samples were used in this study. Of them, 16 were from dengue-infected patients, and eight were from healthy individuals. The Laboratory of Flavivirus from Oswaldo Cruz Institute-FIOCRUZ (Rio de Janeiro, Brazil) provided positive controls, and the infection was confirmed by viral isolation and/or RT-PCR, and seroconversion of the IgG antibody obtained from different health centers, public hospitals, and private clinics throughout the country. Healthy individuals’ (negative control) sera were obtained from the blood center, HEMORIO (Arthur de Siqueira Cavalcanti State Institute of Hematology, Rio de Janeiro, Brazil).

### 2.3. Apparatus and Measurements

Cyclic voltammetry (CV) and differential pulse voltammetry (DPV) analysis were performed, with a multi potentiostat/galvanostat, eight-channel DropSens (Metrohm, Carabanchel, Madrid, Spain) that can act at the same time as eight independent potentiostats/galvanostats. It is integrated with a DELL notebook, controlled by the DropView 8400 software. Disk screen-printed electrodes (SPEs) (4.0 mm, Ø) with screen-printed gold electrodes (SPGE/220AT), along with a counter electrode made of the same material as the working electrode and a silver reference electrode, were obtained from DropSens. The model-printed gold electrode (SPGE/220AT) was used against a gold electrode and a silver reference pseudo-electrode. All the electrodes were screen-printed on a ceramic substrate (3.4 × 1.0 × 0.05 cm) reference electrode and silver electrical contacts. The characterization of the electrodes was conducted using probe electrochemical with 5 mmol L^−1^ K_4_Fe(CN)_6_/K_3_Fe(CN)_6_ in 0.1 mol L^−1^ KCl solution (pH 7.0). The CV was scanned from a scan rate of 0.025 V S^−1^ and potential ranging from −0.6 to 0.6 V, using 6 cycles of scans; greater stability of the SGPE and DPV was detected from a scan rate of 0.01 V S^−1^ and potential ranging from −0.3 to 0.5 V (pulse amplitude, 25 mV; pulse period, 100 ms).

### 2.4. IgG Epitope Mapping

The complete sequence of the non-structural protein 1 (NS1; AAT79552) of DENV-3 circulating in Brazil was obtained through access to the National Center for Biotechnology Information (NCBI) protein, https://www.ncbi.nlm.nih.gov/ (accessed on 24 January 2022). This glycoprotein possesses 46–50 kDa, which associates as a dimer to internal and cytoplasmic membranes and is also secreted, as a hexamer, to the extracellular milieu [39].

Microarrays of peptides based on the SPOT-synthesis technology were used to map linear B-cell epitopes using an Auto-Spot Robot ASP-222 (Intavis Bioanalytical Instruments AG, Köln, Germany), as described previously [40]. Ten linear B-cell IgG epitopes were identified using a pool of a random subset (n = 10) of patient sera infected with DENV (Figure S1).

The multiple alignment of the DENV proteins for selecting the cross-reactive and specific epitopes was performed using the algorithm clustalW2 (Biological sequence alignment editor for Windows 10/11; BioEdit version 7.2), available at https://thalljiscience.github.io/ (accessed on 15 February 2022). The epitope/peptide DENV/18 (SFIID GPNTEPEK) was used in this study.

### 2.5. Solid Phase Peptide Synthesis

The universal (DENV-1-4) epitope peptide DENV/18 (SFIIDGPNTEPEK) was chosen and synthesized according to the standardized procedure [41] using the F-moc strategy and an automatic synthesizer (MultiPep-1, CEM Corp, Charlotte, NC, USA). A polymeric resin (Fmoc-PEG Biotin NovaTag^®^; Sigma-Merck, St Louis, MO, USA) was used, containing a free amino group for binding amino acids associated with biotin. The resin was previously solvated in N, N′-Dimethylformamide (DMF); then, for the construction of the peptide sequence, Fmoc-amino acids were solubilized with the coupling reagents N, N′-diisopropyl carbodiimide (DIC) and Hydroxy benzotriazole hydrate (HOBt), diluted in DMF, and poured into the syringe containing the polymeric support. Between each coupling step, the amino acids were treated with 4-methylpiperidine diluted in DMF to remove the F-moc groups in their N-terminal regions, making them viable for the reaction. In addition, an acetylation process was performed in the amino-terminal region, using acetic anhydride diluted in DMF to avoid the construction of truncated sequences. After the assembly of the peptide sequences, the F-moc groups were removed. The peptide resin was cleaved and fully deprotected using TFA/H2O/EDT/ TIS (94/2.5/2.5/1.0, *v*/*v*) for 90 min. The biotinylated peptide was then precipitated by adding chilled diethyl ether and centrifugation (30,000× *g*, 10 min at 4 °C). The resulting pellet was dissolved in aqueous AcOH (10% *v*/*v*), dried, and stored as a lyophilized powder. It was dissolved in water and centrifuged (10,000× *g*, 60 min at 15 °C) when required. The supernatant was filtered through a Centricon™ (Merck Millipore, Burlington, MA, USA) ten filter, and their identities were confirmed by MS (MALDI-TOF or electrospray).

The peptide concentration was estimated using the ExPASy ProtParam tool at 205 nm using a molar extinction coefficient (http://www.basic.northwestern.edu/biotools/proteincalc.html; Accessed on 20 March 2023) previously defined. The specificity of the DENV/18 was previously determined by an enzyme-linked immunoassay (ELISA) that was standardized previously in our laboratory.

### 2.6. Construction of Biosensor for DENV

The peptide immobilization was conducted using an aqueous solution of 1 m mol L^−1^ L-cysteine (L-Cys) containing glutaraldehyde (GA) 2.5% *w*/*v*. A total of 10 μL of L-Cys and 10 μL of GA were added to each working electrode, oven-dried at 37 °C for one h, and then 20 μL of DENV/18 (0.1 mg mL^−1^) was added. It was refrigerated at about 4 °C overnight.

After immobilization of the peptide-DENV, the SPGE sensors were thoroughly washed with a PBS buffer solution, and then 20 μL of human serum dilutions (1:10; 1:50; 1:100; 1:500, and 1:1000 in PBS pH 7.4 buffer) were added. Tests were carried out with positive samples, i.e., human serum from patients who had dengue, and also analyzed with negative samples, such as human serum from individuals who never had dengue. A parallel analysis was carried out in a study without the presence of serum, substituting this with the addition of PBS buffer pH 7.4, considered the “blank” analysis, and the electrode was incubated for 1h at 37 °C. Afterward, the washing steps were repeated twice, followed by the addition of the probe electrochemical K_4_Fe(CN)_6_/K_3_Fe(CN)_6_ 5 mmol L^−1^ in 0.1 mol L^−1^ KCl solution (pH 7.0).

### 2.7. Analytical Construction of the Biosensor Dengue

The analytical interpretation of the biosensor dengue was measured through incubation with 100 µg mL^−1^ DENV/18 at different dilutions of human serum, which were diluted in PBS (0.1 mol L^−1^, pH 7.4). CV and DPV monitored the biosensor’s performance in an assay using 5 mmol L^−1^ K_4_Fe (CN)_6_/K_3_Fe (CN)_6_ in a 0.1 mol L^−1^ KCl solution (pH 7.0).

The detection of anti-DENV with affinity to the DENV/18 peptide in the patient’s serum was standardized using the decrease in the current signal in the CV and DPV measurements. The difference in the current of the anodic peak between the sera dilution curve of dengue-infected patients and non-infected patients was subtracted from the curve with blank, i.e., only solution PBS. This delta (∆I) was calculated and used to evaluate the analytical responses. All the measurements were carried out in triplicate.

### 2.8. Morphology Characterization

Field emission scanning electron microscopy (FE-SEM) and atomic force microscopy (AFM; Hitachi, Tokyo, Japan) were employed to examine the morphology change of gold working electrodes before and after the modification of DENV/18 peptides. For the SEM analysis, photos were taken on the FlexSEM 1000 II VP-SEM (Hitachi, Tokyo, Japan) at an accelerating voltage of 10 kV and a working distance of 10 mm.

The atomic force microscopy (AFM) analysis was performed at room temperature in air mode using the Nanosurf Flex-Axiom AFM (Nanosurf Inc., Santa Barbara, CA, USA) and in tapping mode using Tap190Al-G cantilever. The size of the analyzed area was 50 × 50 μm. The images obtained were treated and analyzed using Gwyddion 2.51 software.

## 3. Results

### 3.1. Epitope Mapping and Structural Localization of the Biosensor in Detecting DENV

A complete SPOT-synthesis analysis identified 10 linear B-cell IgG epitopes recognized by a pool of patient sera (n = 10) in the NS1 DENV-3 protein (Figure S1). The primary goal of this study was to identify antigenic determinants that could differentiate DENV infections from closely related pathogens, so bioinformatics was used to BLAST the sequence of each of the 10 epitopes. One epitope that originated from the protein NS1 was determined to meet the criteria for a high potential to avoid cross-reactivity with another organism based on the absence of multiple sequential amino acids that were identical to segments in other pathogens, including the most highly similar virus, ZIKA, WNV, and CHYV. This epitope was common to DENV1-4 (Figure S2) and was chosen for further analysis.

The electrochemical biosensor study was performed in parallel with an ELISA-peptide to validate the peptide effectiveness.

**Figure 1:**
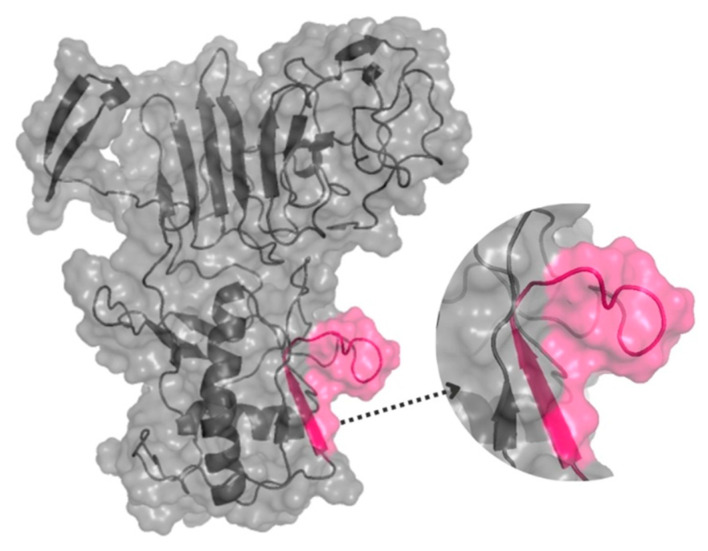
The three-dimensional structure of the NS1-DENV-3 (AAT79552) protein, with the position of the DENV/3 epitope, was identified through SPOT synthesis. The epitope is pink within a model constructed using predicted protein structures I-TASSER. The image was created using PyMol.

The spatial location of the epitope in the tertiary structure of NS1-DENV3 was predicted using the D-I-TASSER server and visualized by the PyMol program version 3.0 (Figure 1). The structural prediction demonstrates that the epitope is on the protein surface and accessible to the solvent. In addition, its largest portion is in the coil/loop region.

### 3.2. Biosensor Design

**Figure 2:**
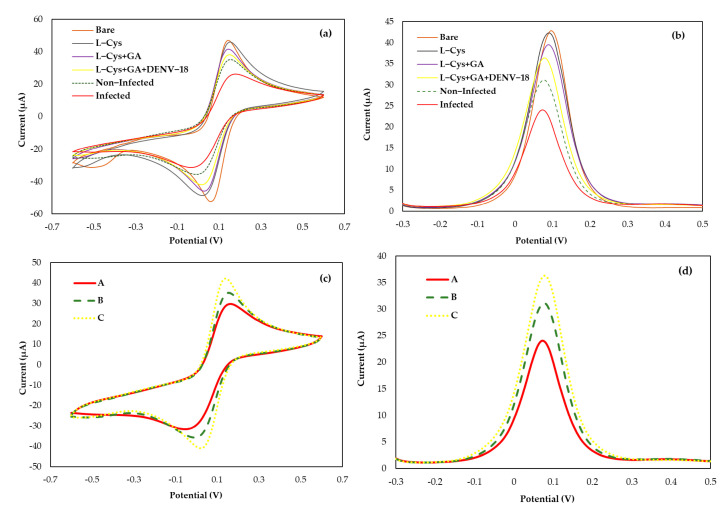
Cyclic voltammograms (**a**,**c**) and differential pulse voltammetry (**b**,**d**) of the biosensor immobilized with the DENV/18 peptide: In (**c**,**d**) (Curve A) patient’s sera infected for DENV; (Curve B) non-infected human serum; (Curve C) buffer solution PBS.

The biosensor design to capture circulating anti-DENV antibodies in patient serum was developed using a screen-printed electrode with a gold working electrode, a gold counter electrode, and a silver reference electrode. Figure 2 shows the electrochemical analyses (a) CV and (b) DPV at each step of the DENV/18 peptide immobilization process, as well as its interaction with human serum.

Due to the presence of the thiol group and its affinity for gold, an aqueous solution of L-cysteine was added to the surface of the working electrode by dripping in two coatings for better fixation on the gold surface. In this case, the L-cysteine solution prepared in aqueous medium forms a passive layer on the electrode surface, thus reducing its active area [42,43], which leads to a slight decrease in the current observed in the voltammograms, as shown in Figure 2a,b. The glutaraldehyde crosslinker was placed after the addition of L-Cys to immobilize DENV peptides by their amine groups, forming a crosslink between the L-Cys amino acid and the amine group of the peptides through the bifunctional aldehyde moieties of GA. As observed in Figure 2a,b, in each step of peptide immobilization on the surface of the working electrode, there is a reduction in the active area of this electrode, tending to slight decreases in the current peaks when compared to the bare gold electrode.

The interactions of DENV/18 with antibodies against DENV circulating in human serum were analyzed from the decrease in current peaks due to greater interaction between the epitopes and the antibodies present in the serum of patients infected with dengue, which caused a blockage on the area of the working electrode measured from the electrochemical probe with ([Fe(CN)_6_]^3−^/^4−^) solution. In the foul of specific DENV antibodies (negative samples), it is observed that the electrical current signal is nearly constant, and the blank is owing to the non-interactions between the peptide DENV/18. The electrochemical biosensor studies were compared to the DENV peptide ELISA (data not shown) to validate the data acquired by the ELISA technique.

### 3.3. Operation Analysis Biosensor

The biosensor operation was estimated in response to the positive and negative samples in serum human for DENV, using a fixed concentration of immobilized DENV/18 peptides on the electrode surface (100 µg mL^−1^). Analytical responses were generated by the differences in the current anodic peaks (at 0.025 V/s scan rate) of CVs and (at pulse amplitude 0.025 V) of DPV before and after the serum addition. Figure 2c,d shows an analysis of typical (c) CV and (d) DPV, with samples 1:50 diluted in PBS, in which the solid line corresponds to the curve of patients infected with dengue (Curve-A); the dashed line corresponds to the curve of non-infected individual response (Curve-B), and the dotted line corresponds to the analysis blank, i.e., only buffer solution PBS (Curve-C).

**Figure 3:**
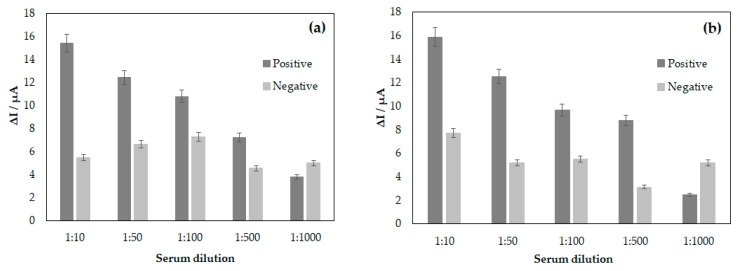
Correlation between serum dilutions for DENV/18 peptide (904-SFIIDGPNTPEC-916) in the analyses between patient’s sera infected with DENV and non-infected human serum, (**a**) analysis by CV and (**b**) DPV.

The analyses of the interactions between DENV/18 peptide (epitope) immobilized on the surface of the electrodes and circulating anti-IgG against dengue in the serum of patients infected with DENV were performed based on the decrease in the current peak maximum measured at the oxidation potential of the redox probe [Fe(CN)^6^]^4−/3−^. A blank was also performed using only PBS buffer solution, and from this blank, the ∆I was calculated for each dilution studied. Figure 3 shows the difference in current obtained between the sera of patients with infection (positive) and without infection (negative) for dengue. According to Figure 3, a greater ∆I can be observed in the lowest serum dilution of 1:10, while with a higher serum dilution analyzed at 1:1000, the ∆I was smaller since the difference between the blank and the diluted solution is smaller. In the analyses, the maximum difference was obtained at the serum dilution of 1:10 for the positive and negative samples; in both cases, in the CV and DPV analyses, the difference in the mean current value reached 15.38 μA in the CV analysis and 15.89 μA using the DPV technique. Meanwhile, a minimum current value considered safe for the analysis was reached at 7.21 μA for CV and 8.79 μA in DPV, for the serum dilution of 1:500. Although there was a considerable difference between the positive and negative samples up to serum dilutions of 1:500, there was no statistically significant difference between the amperometric signals of DENV-positive and negative samples at 1:1000 (3.78 μA for CV and 2.45 μA in DPV, in positive samples and 4.98 μA for CV and 5.16 μA in DPV, in negative samples), i.e., at this dilution, it was not possible to differentiate between positive and negative samples (paired *t*-test, *p* < 0.05). The error bar in Figure 3 represents the standard deviations for three replicate measurements.

### 3.4. Limit of Detection and Specificity of the Biosensor

The detection limit for the bioassay was analyzed by keeping a constant concentration of the DENV/18 peptide (100 µg mL^−1^) fixed on the SPGE surface. The synthetic (904-SFIIDGPNTPEC-916) contains the critical epitope, the antigenic determinant for the detection of antibodies in human serum for dengue, which was immobilized to the area of the working electrode along with a solution L-Cys and the crosslinker solution of GA. A smaller current value can be observed from the analysis of voltammograms, as shown in Figure 2c,d Curve-A; study with a serum of DENV-infected patients. As the interaction between the DENV/18 peptide and human antibodies in the positive samples increases, the blockage of the passage of electric current on the electrode surface will be greater. Consequently, measurements performed using the ([Fe(CN)_6_]^3−^/^4−^) solution electrochemical probe will detect a smaller current.

The same analysis was performed in Figure 2c,d Curve-B. The samples studied in this trial were from an individual not infected by dengue, i.e., in the absence of IgG specific for DENV in human serum, thus generating a greater current signal. Figure 2c,d Curve-C shows the analyses performed without human serum using only PBS buffer solution. This study aimed to analyze some reactions that could ensue merely in the presence of the buffer solution and compare them with the analysis performed with serum free of dengue-specific antibodies.

After the electrochemical study of the interactions of the peptides with human serum samples from patients with dengue, the detection limit analysis for DENV-specific antibodies was performed. Figure 3a,b shows the bar graph constructed from the ΔI calculated by subtraction of the maximum anodic current value between human serum samples and the blank, i.e., PBS solution, for the following dilutions: 1:10, 1:50, 1:100, 1:500, and 1:1000. From this graph, it can be observed that the highest possible dilution for the analysis in this study was 1:500. However, when increasing the dilution of human serum, it was found that there was no longer coherence between the values obtained for patients infected with DENV and those not infected. Therefore, by maintaining a fixed concentration of the EP-DENV peptide 100 µg mL^−1^ in the SPGE, the assay’s detection limit decreased with the increase in the dilution of the serums. Therefore, the ideal dilution obtained in this study was 1:500; above this value for dilution, it is impossible to guarantee that the assay’s specificity will be achieved.

**Figure 4:**
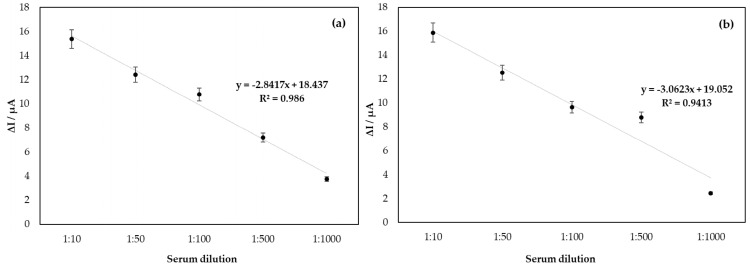
Linearity of analysis of the biosensor for DENV/18 peptide, in the function of serum dilution with antibodies for DENV (**a**) analysis by CV and (**b**) DPV.

Figure 4 shows the linearity curve of the biosensor for DENV developed in the study; based on the model of the immunocomplex theory, DENV/18 peptide, and IgG circulating in human serum from patients infected with dengue, the profile of the curve obtained was linearized for serum dilutions in PBS of 1:10, 1:50, 1:100, 1:500, and 1:1000. The results show an increase in the current response proportional to the serum concentrations, indicating a good analytical adjustment performance (R^2^ = 0.9860 in CV and R^2^ = 0.9413 in DPV; n = 5). The less diluted the serum is, the higher the value in the current delta among the sera analyzed. Therefore, when reaching an optimal serum dilution concentration, the current tends to remain constant as a function of the serum dilution. This can be better observed from the error bar.

Considering the most appropriate dilution factor for the analysis under study, 1:500, the line equation for this concentration was re-adjusted. Figure S4 shows the sensitivity obtained in the assay: 2.62 × 10^−3^ µg mL^−1^ in CV and 2.42 × 10^−3^ µg mL^−1^ in DPV.

Due to the linearity of the analyses, the straight-line slope method was used to calculate the detection limit of the LOD system under study. Using the standard deviation in the analysis with the blank 1.056 in CV and 0.3448 in DPV, the detection limit obtained in the assay was 1.21 ng mL^−1^ in CV and 0.43 ng mL^−1^ in DPV.

### 3.5. Repeatability and Stability of the Biosensor Response

To study the repeatability of the biosensor, serum-DENV/18-GA-L-Cys, four independent electrodes were prepared on different days using 100 µg mL^−1^ of the DENV/18 peptide. The same dilution of sera from DENV-positive patients (1:50) was added to each electrode, followed by the potassium ferrocyanide/ferricyanide ([Fe(CN)_6_]^3−/4−^) electrochemical probe, for the CV and DPV analyses. The four electrodes exhibited similar current responses, with a calculated relative standard deviation of 8.04% in CV and 4.06% in DPV. The calculation data and curves (Figure S5) obtained for each analysis, CV and DPV, are provided in the Supplementary Material. All the solutions and dilutions used in the experiment were prepared on the same day as the analysis. No electrode stability study was performed after the addition of the serum dilution.

### 3.6. Morphological Analysis of the Biosensor

**Figure 5:**
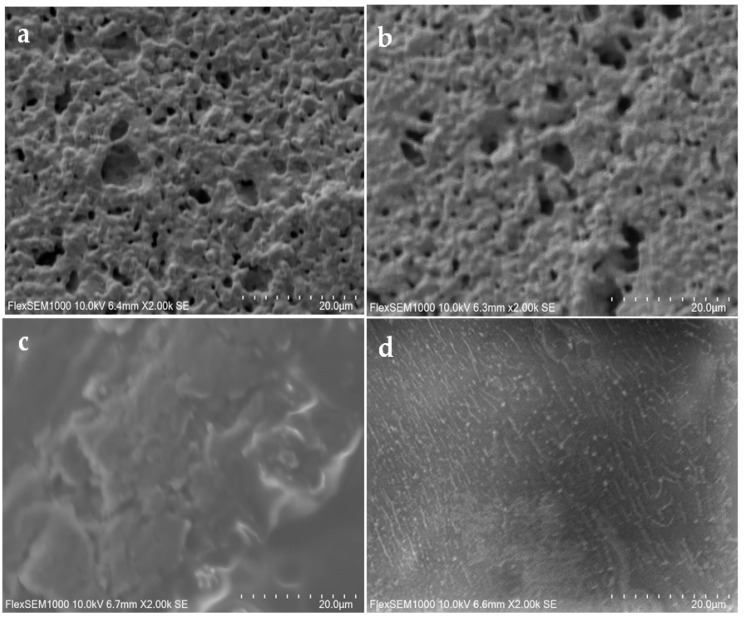
Surface scanning electron microscopy (SEM) analysis of the screen-printed gold electrode–SPGE; (**a**) bare, (**b**) L-Cys-coated gold electrode, (**c**) SPGE with L-Cys/GA, and (**d**) SPGE with L-Cys/GA/ DENV/18 peptide.

The three-dimensional images of the surface of the printed electrode were performed by surface scanning electron microscopy (SEM) analysis to monitor the adsorbed peptide [44]. Figure 5 shows the bare gold electrode (a) and with L-Cys solution (b). Small alterations were observed on the electrode surface with L-Cys solution. On the other hand, with GA solution (c), significant changes were observed, and a greater electrode coating occurred. Figure 5d shows that the L-Cys/GA/DENV/18 peptide was fixed onto the surface of the gold electrode, and its linear structures can be noted in the image.

Atomic force microscopy (AFM) analyses were also performed to analyze the DENV/18 peptide immobilization morphology on the SPGE surface; this characterization technique has been applied in several studies, such as phase changes, precipitate formation, and sensitization processes [45].

**Figure 6:**
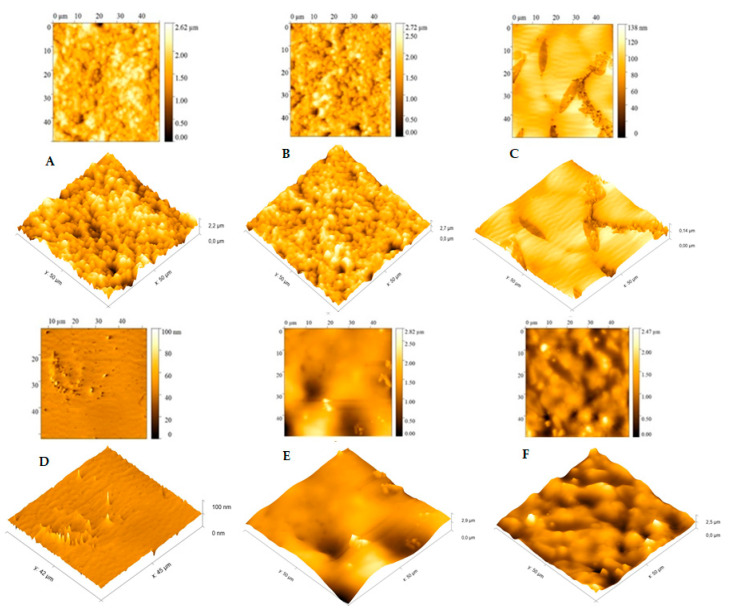
Atomic force microscopy (AFM) analysis and 3D images performed on the working electrode of the SPGE: (**A**) bare gold electrode, (**B**) L-Cys-coated gold electrode, (**C**) L-Cys/GA, (**D**) L-Cys/GA/DENV-18 peptide, (**E**) L-Cys/GA/DENV-18 peptide/human serum DENV-infected, (**F**) L-Cys/GA/DENV-18 peptide/human serum not infected with dengue.

From the data observed in Figure 6, we can analyze each stage of the electrode modification carried out in this study. In the bare gold electrode (Figure 6A), a high average roughness can be observed on the surface of the pure electrode, which shows agreement with the results of the SEM analysis (Figure 5a); in this case, this roughness is due to the cavities present in the SPGE working electrode. As can be seen in Figure 6B, only with the L-Cys solution, practically no change was observed on the electrode surface; the presence of cavities in the SPGE working electrode can also be observed here. Again, this result is corroborated by Figure 5b.

Nonetheless, significant changes on the electrode surface were observed in the presence of the L-Cys plus GA solution (Figure 6C). A greater coating of the electrode, with an apparent complete coverage of the gold electrode’s cavities, and a small film formation is evident. The average roughness in this situation was small, again in agreement with Figure 5c. In the presence of the DENV/18 peptide solution (Figure 6D; L-Cys/GA/ DENV-18 peptide), a more pronounced decay in the average roughness of the SPGE is depicted, indicating that the peptides’ fixation occurred on the electrodes.

The AFM analysis made it possible to better observe the interaction of the DENV/18 peptides with the antibodies for dengue circulating in the human serum. The study was performed with a serum dilution of 1:50. The results obtained for this analysis were more conclusive than the results of the SEM analysis, where a better interaction of the DENV/18 peptide with human serum could be observed using this technique. In Figure 6E, L-Cys/GA/DENV/18 peptide/human serum DENV-infected, the coating on the electrode surface was more significant because the relative roughness was lower when compared to Figure 6F, L-Cys/GA/DENV/18 peptide/ human serum not infected with dengue; in this case, the relative roughness was higher, corroborating the results obtained in the CV and DPV analyses, in which, in the absence of dengue-specific antibodies, the interaction with the DENV-18 peptide is lower.

## 4. Discussion

The search for rapid and sensitive diagnostic tests for various pathologies is still challenging in the clinical area. Dengue is a viral infection widely spread by the *Aedes aegypti* mosquito with a high transmission and lethality rate [46,47,48]. The diagnosis of dengue is still a problem since most of it is made from the NS1 protein, which presents cross-reactions with other flavivirus family members. Therefore, diagnostic tests without present cross-reactions are important for control programs, as they determine which specific actions should be taken. Electrochemical biosensors are constructed as tools with high sensitivity, low costs, rapid results, easy portability, and the need for only small sample volumes [49]. In this study, we developed a sensitive electrochemical immunoassay efficient of diagnosing dengue using a selected epitope, the DENV/18 peptide (SFIIDGPNTPEK), derived from the NS1-DEN-3 protein of DENV. This peptide was chosen from a set of six epitopes identified by a microarray assay. It was shown to be an immunodominant epitope with a specificity of 100% and sensitivity of 98% in an ELISA against a panel of 84 sera from patients with various diseases [Figure S3].

The antigen is key to providing high sensitivity and specificity in an immunoassay. This study used the EP-DENV epitope as an antigen to capture circulating DENV antibodies in blood serum. The developed immunoassay was combined with an SPE-based platform that used the potassium ferrocyanide/ferricyanide electrochemical probe to detect the redox signal generated in the analysis, improving costs and portability.

The performance of the biosensor to detect DENV in sera from infected patients showed high selectivity when compared to sera from healthy (uninfected) individuals, also exhibiting a detection limit of 100 µg mL^−1^ for the synthetic peptides, and 1.21 ng mL^−1^ in CV and 0.43 ng mL^−1^ in DPV for human serum. The mechanism of the biosensor depends on the interaction of the EP-DENV peptide with DENV antibodies, observed by the decrease in the current measured at the active electrode during CV and DPV due to the blockage of the current passage at the working electrode. The good reproducibility and sensitivity exhibited by the biosensor indicate that this methodology can be practical and usable. Typically, only 20 s are required for measurements of the electrochemical methods, compared to about 10 min for the spectrophotometric method and 3 h for an ELISA, both of which require sample preparations. This methodology, which uses peptides immobilized on electrode surfaces for constructing different biosensors, is quite consolidated in the literature, Table 1 presents some types of biosensors and their applicability, as reported in the literature, and the biosensor developed in this study.

## 5. Conclusions

In this study, we designed a sensitive sensor capable of measuring DENV1-4 reactive antibodies in sera of infected patients using the specific epitope peptide DENV/18 (SFIIDGPNTPEK). The developed biosensor exhibited good selectivity when compared to sera from (non-infected) healthy individuals, also exhibiting a limit of detection of 100 µg mL^−1^ for the synthetic peptides and 1.21 ng mL^−1^ in CV and 0.43 ng mL^−1^ in DPV for the human serum. The sensor developed here proved useful and had a high potential to be miniaturized. In addition, it provides a platform for rapid and sensitive detection with high specificity and sensitivity to the rapid serological diagnostic test for dengue.

## Figures and Tables

**Table 1:** Types of biosensors that use peptide as a detection method.

Protein	Electrode + Modification	Peptide Sequence	Method	Signal Probe	LOD	Ref.
Casein Kinase II	MSF_S_/ITO	NH_2_- RRRRRRRRRRRADDSDDDDD-COOH	DPV	Ferrocene	0.095 U mL^−1^	[50]
Anti-p24	AuE/SAM	HS–(CH_2_)_11_-EAAEWDRVHP-K-MB HS–(CH_2_)_11_-SGSGSGEAAEWDRVH P–K-MB HS–(CH_2_)_11_EKEKEKEAAEWDRVHP-K-MB	CV	Methylene blue	0.5 nM 1 nM 1 nM	[51]
Cardiovascular biomarker B-type	SPCE/4- Aminothiophenol + AuNPs	H_2_N-Phe-SPCE	EIS, CV	Horseradish peroxidase	4 pg mL^−1^	[52]
Cardiac troponin I	GCE + AuNPs + PEG/SAM	CFYSHSFHENWPS	EIS– signal-on	Label-free	3.4 pg mL^−1^	[53]
OMPA_RICRI (H6PGA4)	SPCE/SAM+ anti-huIgG-AP	846ANVVLFNDAVQLTQ859	CV	Hydroquinone diphosphate	0.01 µg mL^−1^	[54]
NS1-DENV-3	SPGE/L-Cys + GL	SFIIDGPNTEPEK	CV, DPV	Potassium ferrocyanide/ferricyanide	1.21 ng mL^−1^ 0.43 ng mL^−1^	This work

## Data Availability

The data presented in this study are available upon request from the corresponding author.

## Supplementary Materials

The following supporting information can be downloaded at https://www.mdpi.com/article/10.3390/bios15040236/s1, Figure S1: IgG epitope mapping of DENV-3 NS1 protein. A membrane-bound peptide library representing the NS1 was probed with a pool of patient’s sera (n = 10), and reactivity was detected using goat anti-human IgG alkaline phosphatase labeled secondary antibody and chemiluminescence substrate. Panels (A) present an image of the peptide array showing reactivity as dark circles. The panels in (B) show the list of epitopes identified in DENV-3 NS1 through SPOT synthesis, and in (C), the list of synthesized peptides. Figure S2: Blast cross-reactivity of DENV-3 NS1 epitope assessed by database search. Figure S3: Reactivity of serum from DENV-infected patients (several serotypes) against the synthetic peptide by an in-house ELISA. Figure S4: Linearity of analysis of the biosensor for DENV/18 peptide, in the function of serum dilution with antibodies for DENV (a) analysis by CV and (b) DPV. Calculations on sensitivity from the straight-line equation for dilutions are considered reliable in the study. Calculation of LOD of some biosensors using synthetic peptides. Figure S5: Repeatability of analysis of the biosensor for DENV/18 peptide, for the serum dilution with antibodies for DENV of 1:50 (a) analysis by CV and (b) DPV. Calculation of relative standard deviation for the analyses.
