# Supplementary material for: Ultrasensitive Peptide-Based Electrochemical Biosensor for Universal Diagnostic of Dengue
Source: Biosensors (Basel). 2025 Apr 8;15(4):236. doi: 10.3390/bios15040236 (PMC12024919; doi:10.3390/bios15040236)
Supplement: Supplementary file 1 [file biosensors-15-00236-s001.zip › biosensors-3355055-supplementary.pdf]

A

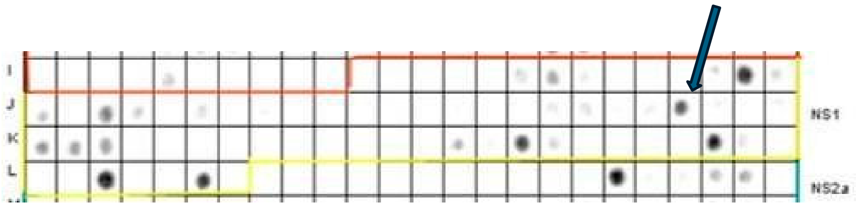

B

	721	731	741	751	761	771	
	|	|	|	|	|	|	
721						DMGCVI N	780
781	WKGKELKC GS	GIFVTNEVH T	WTEQYKFQ AD	SPKRVATAI A	GAWENGV CGI	RSTTRMENL L	840
841	WKQIANELN Y	ILWENDIKL T	VVVGDITGV L	EQGKRTLTP Q	PMELKYSW KT	WGLAKIVTA E	900
901	TQNSSFIIDG	PSTPECPSAS	RAWNVWEV ED	YGFGVFTTN I	WLKLREVY TQ	LCDHRLMSA A	960
961	VKDERAVH AD	MGYWIESQ KN	GSWKLEKAS L	IEVKTCTWP K	SHTLWSNG VL	ESDMIIPKSL	1020
1021	AGPISQHNH R	PGYHTQTA GP	WHLGKLEL DF	NYCEGTTVV I	SENCGTRG PS	LRTTTVSGK L	1080
1081	IHEWCCRSC T	LPPLRYMGE D	GCWYGMEI RP	INEKEENMV K	SLASA		

Figure S1. IgG epitope mapping of DENV-3 NS1 protein. A membrane-bound peptide library (6G peptides with 15 aa long and overlapping with 10 residues) representing the NS1was probed with a pool of patients (n=10), and reactivity was detected using goat anti-human IgG alkaline phosphatase labeled secondary antibody and chemiluminescence substrate. The panels in (A) present an image of the peptide array, showing reactivity as dark circles. The panels in (B) show the localization of the epitopes identified in DENV-3 NS1 through SPOT synthesis in the protein's primary structure. The arrow shows the epitope in the cellulose membrane.

Query	895KIVTAETQNSSFIIDGPSTPECPSASRAWNVWE		927	Organism
Q99D35.1	895..........SFIIDGPSTPEC		927	Dengue 3
P27915.1	895..........SFIIDGPSTPEC		927	Dengue 3
Q5UB51.1	895..........SFIIDGPNTPEC		927	Dengue 3
Q6YMS3.1	895..........SFIIDGPNTPEC		927	Dengue 3
Q6YMS4.1	895..........SFIIDGPN....		927	Dengue 3
P17763.2	897..IG.DV..TTFIIDGPN....	.DNQ..<br>..	I..929	Dengue 1
P27909.2	897..IG.DI..TTFIIDGPD....	.DEQ..<br>..	I..929	Dengue 1
P33478.2	896..IG.DI..TTFIIDGPD....	.DDQ..<br>..	I..928	Dengue 1
P29991.1	897.MLST.SH.QT.FIDGPE.A...	NTN...<br>.	SL.929	Dengue 2
Q2YHF2.1	896..F.P.AK..T.LIDGPD.S...	NER...<br>.	FL.928	Dengue 4
Q58HT7.1	896..F.P.AK..T.LIDGPD.S...	NER...<br>.	FL.928	Dengue 4
Q5UCB8.1	896..F.P.AR..T.LIDGPD.S...	NER...<br>.	FL.928	Dengue 4
P09866.2	896..F.P.AR..T.LIDGPD.S...	NER...<br>.	SL.928	Dengue 4
P14337.2	897.MLST.SH.QT.LIDGPE.A...	NTN...<br>.	SL.929	Dengue 2
P07564.2	897.MLST.SH.QT.LIDGPE.A...	NTN...<br>.	SL.929	Dengue 2
P14340.2	897.MLST.SH.QT.LIDGPE.A...	NTN...<br>.	SL.929	Dengue 2
P29990.1	897.MLST.SH.QT.LIDGPE.A...	NTN...<br>.	SL.929	Dengue 2
P27914.1	617.MLST.LH.QT.LIDGPE.A...	NTN...<br>.	SL.649	Dengue 2
P12823.1	897.MLST.LH.QT.LIDGPE.A...	NTN...<br>.	SL.929	Dengue 2
Q9WDA6.1	897.MLST.LH.QT.LIDGPE.A...	NTN...<br>.	SL.929	Dengue 4
P30026.1	897.M.PT.PH.QT.LIDGPE.A...	NTN...<br>.	SL.929	Dengue 4

Figure S2. Blastp analysis of cross-reactive peptides to DENV 3 epitope SFIIDGPSTPEC for NSP1 protein.

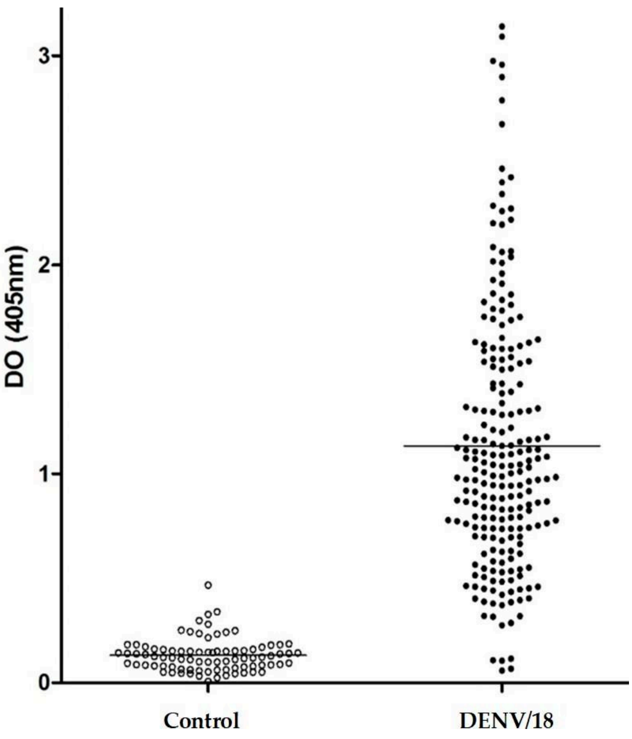

Figure S3. Reactivity of serum from DENV-infected patients (several serotypes) against the synthetic peptide (PEG-biotin-SFIIDGPSTPEC) by an in-house ELISA. Each circle corresponds to an individual serum sample. The horizontal bars in each group represent the media for each peptide. A serum from goat anti-human IgG alkaline phosphatase conjugated and pNPP as a substrate. Control is normal, healthy individuals' sera (n=85), and DENV/18 is the sera of dengue patients (n=221).

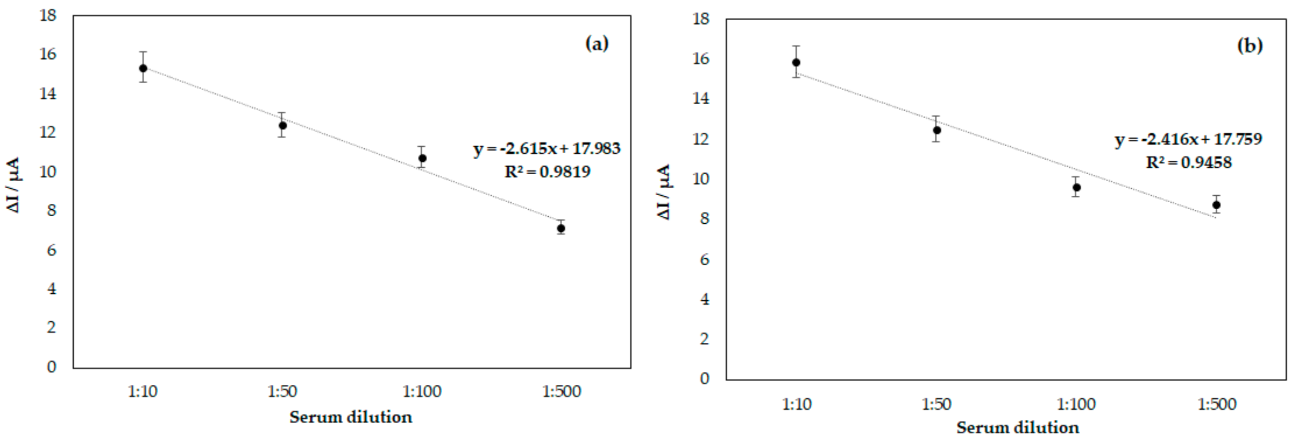

Figure S4. Linearity of analysis of the biosensor for DENV/18 peptide, in the function of serum dilution with antibodies for DENV (a) analysis by CV and (b) DPV. Calculations of sensitivity from the straight-line equation for dilutions are considered reliable in the study. Calculation of LOD of some biosensors using synthetic peptides.

Considering the most appropriate dilution factor for the analysis under study of 1:500, the line equation for this concentration was re-adjusted. In CV y= -2.615x + 17.983 and in DPV y= -2.416x + 17.759, the detection sensitivity was 2.62 x 10^{-3} µg mL^{-1} in CV and 2.42 x 10^{-3} µg mL^{-1} in DPV.

Due to the linearity of the analyses, the straight-line slope method was used to calculate the detection limit of the LOD system under study.

$$LOD = \frac{3*\delta_{(blank)}}{\text{sensitivity}}$$

Considering the standard deviation in the analysis with the blank 1.056 in CV and 0.3448 in DPV, the detection limit obtained in the study was 1.2114 ng mL^{-1} in CV and 0.4280 ng mL^{-1} in DPV.

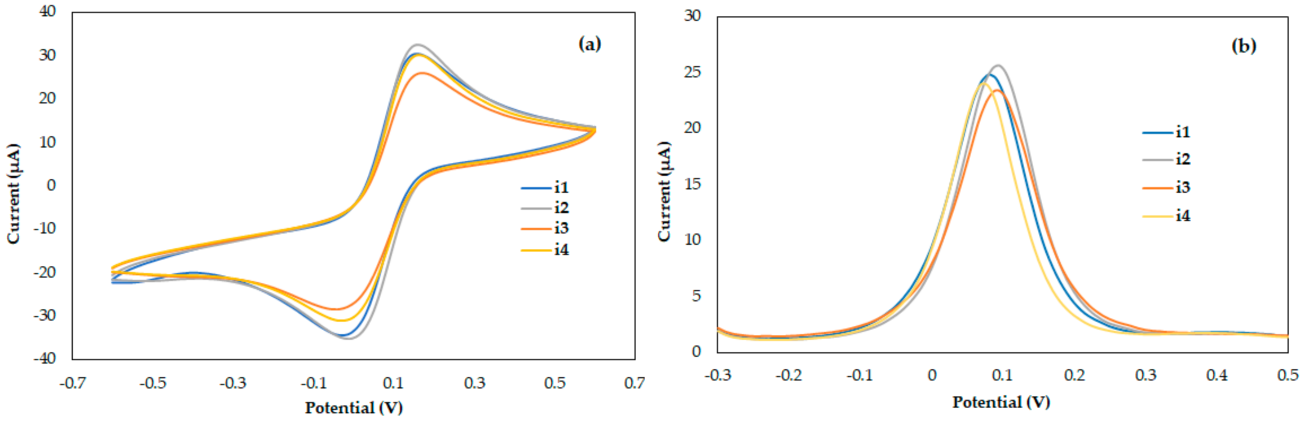

Figure S5. Repeatability of analysis of the biosensor for DENV/18 peptide, for the serum dilution with antibodies for DENV of 1:50 (a) analysis by CV and (b) DPV. Calculation of relative standard deviation for the analyses.

CV(RSD) = $\frac{s}{\bar{x}} \times 100$

<u>Serum dilution 1: 50</u>

Cyclic voltammetry (CV)

$i_1 = 3.0857 \times 10^{-5}$ A

$i_2 = 3.2832 \times 10^{-5}$A

$i_3 = 2.6997 \times 10^{-5}$A

$i_4 = 3.0849 \times 10^{-5}$A

$\bar{\imath} = 3.0384 \times 10^{-5}$ A

$S = 2.443 \times 10^{-6}$

CV = 8.04 %

Differential pulse voltammetry (DPV)

$i_1 = 2.3821 \times 10^{-5}$ A

$i_2 = 2.4825 \times 10^{-5}$A

$i_3 = 2.2775 \times 10^{-5}$A

$i_4 = 2.4817 \times 10^{-5}$A

$\bar{\imath} = 2.4059 \times 10^{-5}$ A

$S = 9.7744 \times 10^{-7}$

CV = 4.06 %
